# Neural correlates of retrieval-based enhancement of autobiographical memory in older adults

**DOI:** 10.1038/s41598-020-58076-6

**Published:** 2020-01-29

**Authors:** Qianli Xu, Jiayi Zhang, Joanes Grandjean, Cheston Tan, Vigneshwaran Subbaraju, Liyuan Li, Kuan Jin Lee, Po-Jang Hsieh, Joo-Hwee Lim

**Affiliations:** 1 Institute for Infocomm Research, Agency for Science, Technology and Research, Singapore, Singapore; 2 Singapore BioImaging Consortium, Agency for Science, Technology and Research, Singapore, Singapore; 3 A*STAR Human-Centric Artificial Intelligence Programme, Singapore, Singapore; 4 Department of Psychology, National Taiwan University, Taipei, Taiwan

**Keywords:** Long-term memory, Human behaviour

## Abstract

Lifelog photo review is considered to enhance the recall of personal events. While a sizable body of research has explored the neural basis of autobiographical memory (AM), there is limited neural evidence on the retrieval-based enhancement effect on event memory among older adults in the real-world environment. This study examined the neural processes of AM as was modulated by retrieval practice through lifelog photo review in older adults. In the experiment, blood-oxygen-level dependent response during subjects’ recall of recent events was recorded, where events were cued by photos that may or may not have been exposed to a *priori* retrieval practice (training). Subjects remembered more episodic details under the trained relative to non-trained condition. Importantly, the neural correlates of AM was exhibited by (1) dissociable cortical areas related to recollection and familiarity, and (2) a positive correlation between the amount of recollected episodic details and cortical activation within several lateral temporal and parietal regions. Further analysis of the brain activation pattern at a few regions of interest within the core remember network showed a training_condition × event_detail interaction effect, suggesting that the boosting effect of retrieval practice depended on the level of recollected event details.

## Introduction

Autobiographical memory (AM) - the memory of events or facts retrieved from an individual’s own life - can be context-specific with rich episodic details, or acontextual with only the gist of self-relevant events or personal knowledge^1–3^. The former is often associated with the retrieval of specific events that can be measured by the amount of recollected event details^4,5^ or the subjective vividness of memories^4–7^; and the later reflects the experience of recognition without recalling details (i.e., familiarity)^8,9^. Another related concept is the temporal specificity that indicates whether an event memory is specific to one point in time or repeated over time^4,10^. The ability to recollect event details is indicative of psychological and cognitive functioning, and are influenced by aging^10–12^. For example, older adults tend to remember fewer episodic details than young adults^5,13^, which in turn contribute to other types of cognitive problems, such as, susceptibility to AM conjunction errors^14^ and difficulties in imagination^15^. Therefore, it is beneficial to maintain higher level of autobiographical event memory.

Many researchers investigated the neurocognitive mechanisms of AM, such as, the brain network that is selectively activated by personal episodic memory versus other non-personal memory (sometimes called the “AM retrieval network”)^16–20^. The characteristics of AM has also been studied according to the dissociable neural structures and processes related to the recall of personal specific events versus general autobiographical events or personal semantic knowledge^2,21,22^. Some studies investigated further into brain regions that tracked the amount and fidelity of recollected information^23–25^. It was shown that the amount of recollected event details modulated the activation in some cortical areas, such as, ventromedial prefrontal cortex (vmPFC)^26,27^, hippocampus, posterior parahippocampal gyrus^28^, and left angular gyrus^27^. Some studies examined the effect of aging on the neural process of AM retrieval, and found a few possible neural markers related to reduced episodic richness among older adults, such as, reduced engagement of hippocampus^29^, reduced involvement of dorsal anterior cingulate cortex^12^, and reduced ability to perform strategic control (mediated by frontal lobes) over the recovery of specific details (mediated by hippocampus)^30^.

Despite the progress in neurocognitive studies on AM, extant work is inadequate in a few aspects. First, although many studies have used photo cues taken in the real world by participants^7,17,20,27,28,31–34^, the experimental paradigms of these studies were notably different in terms of sample sizes, encoding strategies, methods to select visual stimuli, and metrics used to characterize recollective memory (e.g., sense of reliving^26,33^, vividness^35^, autobiographical memory test^10^, etc.), leading to inconsistent findings^17,32^. Second, in view of the importance of AM in mental health and aging, there is limited neuroimaging evidence on how subjects’ performance in the recall of autobiographical events is affected by cognitive intervention. Recent studies showed that more episodic details could be retrieved through retrieval practice as compared to a re-study process, known as the “testing effect”^36,37^. A few studies focused on reactivation-induced updating that enhanced or distorted memory due to exposure to target or lure images in the real world^20,33,34^. However, given the constrained task space (e.g., museum tour) and the resultant lack of intensity of intervention, these studies provided limited insight into how autobiographical event memory could be affected by systematic intervention programs. Some researchers adopted a training paradigm and examined how episodic specificity induction (ESI) - brief training in recollecting episodic details of past experiences - enhanced subjects’ performance in subsequent cognitive tasks that relied on episodic memory retrieval^3,38^. However, the boosting effect was based on the performance disparity in two distinctive types of tasks - one relying on episodic retrieval (e.g., mentalizing future events), and the other not (e.g., semantic object comparison). It is unclear if such an effect persists on similar personal events that differ only in the amount of event details recollected. Third, many evidences on the neural mechanisms of AM were collected from young adults. While some researchers studied age-related effects on the neural correlates of AM retrieval^12,30^, they did not explore the effect of cognitive intervention on the recall of event details. Meanwhile, it was shown that reactivation-induced memory update was reduced in older adults^32^, whereas no neurocognitive basis was established. It is interesting to examine whether recollection of personal events is modulated by cognitive intervention among older adults, and if so, what are the underlying neural mechanisms.

The aim of the current study is to extend the understanding of the neural correlates of AM, focusing on the characterization of neural substrates that track the amount of recollected event details, and how the recollective process is affected by retrieval-based intervention in older adults. To do so, we designed an experiment to collect neuroimaging data along with subjects’ memory of personal experiences at varying levels of episodic details. We adopted the “Remember/Know” paradigm to induce recollection vs. familiarity of events^9,28^, and evaluated the recollective process according to subjective report of recalled episodic details. It should be noted that recollection of event details is just one performance indicator of autobiographical event memory. Other psychometrics include emotion, vividness, personal significance^4,5,39^, temporal specificity^10,^ and sense of reliving^6,34^. These metrics are widely used in neuropsychology as indicators of subjects’ cognitive status, which contribute to the understanding of clinical disorders of memory^3,11,40^. This research focuses on the recollected event details as the key indicator in agreement with recent reports on this topic^7,27,28^. We adopted wearable cameras to collect lifelogging photos and incorporated retrieval-based training into the cycle of memory encoding, updating, and retrieval in the real-world environment. Finally, we conducted the experiment with older adults, who are at higher risk of memory loss, and thus may benefit most from cognitive intervention. This will allow us to find direct evidence on the neural correlates of retrieval-induced enhancement effect among the target population.

We predict that retrieval practice enhances autobiographical event memory by increasing the ratio of events that are recollected with more episodic details (behavioral characteristics). Moreover, we expect to identify a “remember-sensitive network” (thereafter called remember network) that is disproportionally activated by “remembered” events relative to “known” events, in agreement with the core recollection network previously identified in laboratory settings^23,41–44^. Within this network, certain cortical areas are expected to be activated disproportionally by the amount of recollected episodic details (Hypothesis 1). The identified areas, if any, will extend the limited and inconsistent findings on the neural substrates that track the amount of information recollected. We expect to observe such an effect within the left hippocampus and left posterior parahippocampal gyrus^28^, and left angular gyrus^27^. Such an effect has also been observed within the ventromedial and inferior PFC among young adults^26,27^. However, it was postulated that the contribution of these regions to strategic control during AM retrieval was reduced among older adults^12,30^. Therefore, we expect to see reduced involvement of these regions in tracking the amount of event details. It should be noted that this study did not explore age-related differences on the respective neural mechanisms. Therefore, we did not include a control group of young adults to characterize the difference. Finally, extending the constructive episodic simulation hypothesis^15^, we predict that retrieval practice affects memory of past experiences to varying extents depending on the level of recollected details (Hypothesis 2). Specifically, despite possible age-related diminishing of training effect^32^, we expect to observe greater disparity in brain activation with “remembered” relative to “known” events in the trained than the non-trained condition (H2.1). Alternatively, such a disparity may exist when contrasting “remember” at different levels of episodic details (H2.2).

## Method

### Participants

**Figure 1:**
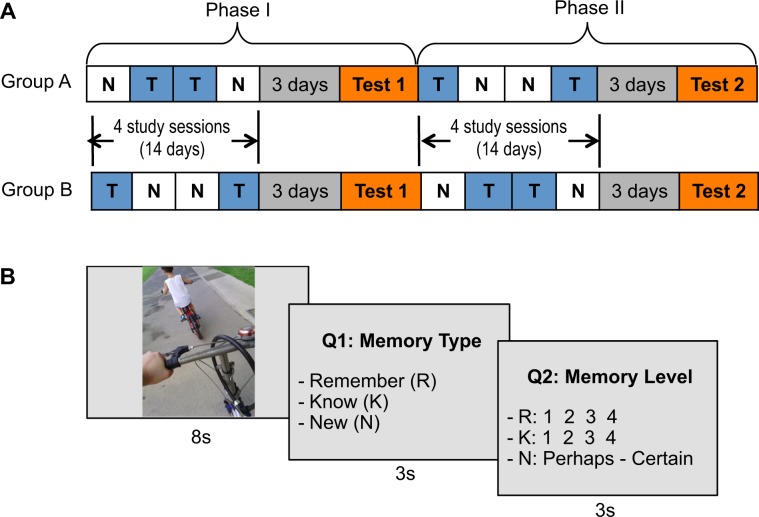
Experiment design. (**A**) Experiment schedule. *T* represents a trained cycle and *N* represents a non-trained cycle. Each *T* and *N* consists of lifelog recording over 3–4 days, followed by a study session of cued-recall or a rest without recall, respectively. In Phase I, after 4 study sessions, a test session is carried out with fMRI scan to test subjects’ recall of recent life. The study-test process is repeated in Phase II whereas the order of *T*-*N* is changed. Subjects are randomly divided into two groups (A and B) with different combinations of *T*-*N* cycles. (**B**) Main steps in a trial of the memory test with fMRI scan. Each trial involved a stimuli presentation (8 seconds) and two questions (3 s each), where Q1 was the type of memory and Q2 was the fine-grained level of the respective memories.

A total of 38 (excluding 5 dropouts) elderly subjects participated in the study (age: 63.1 ± 6.2 years; 26 females; 35 right-handed). None of them had self-reported dementia or cognitive decline. All subjects had normal or corrected to normal vision; could read, speak and write in English. Subjects were randomly allocated to two groups (A: 20 subjects; B: 18 subjects), which differed according to the experiment schedule (Fig. 1A). The study was approved by the Institutional Review Board (IRB) of the National University of Singapore. All methods were performed in accordance with the relevant guidelines and regulations. Informed written consent was obtained from all subjects before the start of the experiment.

### Experiment design

Each subject used a chest-mounted camera which took 2 photos per minute. The duration of experiment was about 9 weeks, consisting of eight study sessions and two test sessions in two phases (Fig. 1A). By including two phases, we wanted to (1) get sufficient samples while avoiding dense sampling of life events that may cause interference in recall tests (refer to Discussion), and (2) ensure the reliability of the results by checking the consistency of outcomes in two phases. In a study session, subjects either engaged in guided recall of life experiences with lifelog photo cues (trained condition) or had a rest without cued-recall (non-trained condition). In a trained condition, the experimenter selected “eventful” photos from the subjects’ recent recording, where “eventful” photos refer to those showing activities and moments that were of personal significance. In a test session, subjects were shown 144 photo stimuli, $\frac{1}{3}$ of which were drawn from the trained condition, $\frac{1}{3}$ from the non-trained condition, and $\frac{1}{3}$ not from the subjects (lure). Note that photos used in training were not re-used as stimuli in testing, but were replaced by similar photos of the respective events. Subjects reported their memory of depicted events in two questions: Q1- memory type (Remember, Know, and New), and Q2 - memory level (recollected event detail 1–4, familiarity level 1–4, and confidence of a “New” judgment) (Fig. 1B). Meanwhile, subjects were scanned for blood-oxygen-level dependent (BOLD) contrast using functional magnetic resonance imaging (fMRI).

### Behavioral analysis

Subjects’ responses to Q1 were categorized into five types. (1) *R* (short for R-hit): own photos correctly recognized as “Remember”, (2) *K* (short for K-hit): own photos recognized as “Know”, (3) *CR*: correct rejection of lure photos as “New”, (4) *WA*: wrong accept of lure photos as “Remember” or “Know”, (5) *Miss*: own photos considered as “New”. With respect to Q2, the following categories were defined for subjects’ own photos. (1) *R*-*II* (strong remember): remember with detail levels 3 or 4, (2) *R*-*I* (weak remember): remember with detail levels 1 or 2, (3) *K*-*II* (high familiarity): know with familiarity levels 3 or 4, (4) *K*-*I* (low familiarity): know with familiarity levels 1 or 2, (5) *M*-*II*: Miss with high confidence (“Certain”), (6) *M*-*I*: Miss with low confidence (“Perhaps”). (See Supplementary Fig. S2 for details of the mnemonic categories). Note that the 4-level memory responses for “Remember” and “Know” were partially collapsed by combining two adjacent levels because the number of responses in a single level of four was small for many subjects. Except for *WA* and *CR* (which originated from lure photos), each of the above categories was classified into trained or non-trained condition, e.g., *R*-*II* under the trained condition is denoted as *R*-*II*_*T*_. The behavioral data and the fMRI data was filtered according to a few *a prior*i exclusion criteria (Refer to Supplementary) to exclude abnormal results caused by subject non-compliance, e.g., inattention, excessive guess work, and head motion, etc. Accordingly, the sample size was reduced to 37 subjects. Behavioral analysis was carried out using a linear mixed model analysis in R (LME4 package v1.1-17). Four factors “Training”, “Scan”, “Group” and “Gender” were modelled as fixed effects and “Subject” as a random effect to account for individual intercepts. Fixed effects significance was tested using general linear hypothesis test (LH-test, two-sided) using the multcomp package (1.4–8). Since the experiment had two scans in two phases, we first examined whether the behavioral performance was affected by the scan order. Based on the general linear hypothesis testing on training condition (trained vs. non-trained) × scan order (scan 1 vs. scan 2), there was an absence of interaction effect on remember (*R*) (LH-test: *z* = −1.18, *p* = 0.240). Nor was there a main effect of scan order on *R*, LH-test: *z* = 0.02, *p* = 0.981. Similar observations were made for the other mnemonic categories (See Supplementary for details). It showed that the results were consistent in two phases. Therefore, the behavioral data from both scans were combined.

### fMRI data acquisition and preprocessing

All MRI data were acquired on a Siemens PRISMA 3 T scanner with a 32 channel head coil. At the start of each session, structural image was collected using a T1mprage sequence with a repetition time (TR) of 2300 ms and a echo time (TE) of 2.22 ms, number of slices (NS) 192, slice thickness (ST) 1 mm, matrix size (MS) 192 × 256. Functional images were collected using axial T2*-weighted gradient-recalled echo-planar imaging and a TR of 2000 ms, TE of 30.0 ms, flip angle 90°, NS 36, ST 3 mm, MS 64 × 64 leading to voxel dimension 3 × 3 mm^2^, and an acceleration factor of 2. A set of 175 volumes aligned to the anterior and posterior commissure was acquired for each acquisition runs. Preprocessing was carried out in BROCCOLI^45^. Functional images were corrected for motion and smoothed with a 5-mm-full-width half-maximum Gaussian filter with normalized convolution kernel. Structural image for each scan session was registered individually to the Montreal Neurological Institute (MNI) 2 mm template using ANTs^46^, and following that the functional images in the corresponding scan were registered to the structural images in the MNI standard space. Individual motion parameters from each individual scan were used as co-regressors for the first-level general linear model (GLM) together with four detrending regressors (intercept, linear, quadratic, and cubic trends) to account for the intercept and drifts. First-level GLM was carried out in BROCCOLI using a boxcar design model convolved with a gamma function.

### Analysis of fMRI data

To identify brain regions that were sensitive to recollection vs. familiarity, seven regressors were generated based on level 1 mnemonics, i.e., *R*, *K*, *CR*, *WA*, *Miss*, *NoResponse*, and *Dummy* regressor. Regressors within a comparison were balanced for the number of events to ensure that statistical significance was not attributed to the number of events. Excess events were randomly assigned to dummy regressors, added as covariates in the model. Next, for analysis related to the recollective memory on event details (H1), four regressors were generated in relation to the recollected event details, i.e., *R*-*II*, *R*-*I*, *NoResponse*, and *Dummy*. As complementary analysis, we also examined possible neural substrates related to the level of familiarity using four regressors: *K*-*II*, *K*-*I*, *NoResponse*, and *Dummy*. Finally, to identify the brain regions that were differentially activated due to training, two tests were conducted: H2.1 - training effect with respect to two memory types using six regressors: *R*_*T*_, *K*_*T*_, *R*_*N*_, *K*_*N*_, *NoResponse*, and *Dummy*, and H2.2 - training effect with respect to recollected event details using six regressors *R*-*II*_*T*_, *R*-*I*_*T*_, *R*-*II*_*N*_, *R*-*I*_*N*_, *NoResponse*, and *Dummy*. We conducted an additional analysis on the training effect with respect to two levels of familiarity using six regressors: *K*-*II*_*T*_, *K*-*I*_*T*_, *K*-*II*_*N*_, *K*-*I*_*N*_, *NoResponse*, and *Dummy*. After applying aforementioned exclusion criteria, a total of 68 scans (32 from Scan 1, 36 from Scan 2) were included. Further balancing the number of events in each regressor, 25 scan sessions were excluded for analyzing level 2 mnemonics. Voxelwise analysis was carried out using nonparametric one-sample *t*-test implemented in BROCCOLI with 5000 permutations together with cluster correction (*p* < 0.05). Following similar process as in behavioral analysis, we checked potential scan order effect with respect to the fMRI data. No scan order effect were observed so that the fMRI data from both scans were combined. Voxelwise analysis was carried out using nonparametric one-sample *t*-test implemented in BROCCOLI with 5000 permutations together with cluster correction. Statistical maps are shown as colour-coded *t*-statistics overlaid on the MNI template. All anatomical references were made with reference to the 2 mm Harvard-Oxford cortical and subcortical structural atlas. Analysis on regions of interest (ROI) (discussed later) were carried out using a linear mixed model analysis similar to behavioral analysis. Least-square regressions were weighted with the number of events in the ROI analysis to mitigate poorer parameter estimation in cases of fewer events. Correction for multiple hypothesis testing was carried out using false discovery rate.

## Results

### Training enhances recollected autobiographical event details

**Figure 2:**
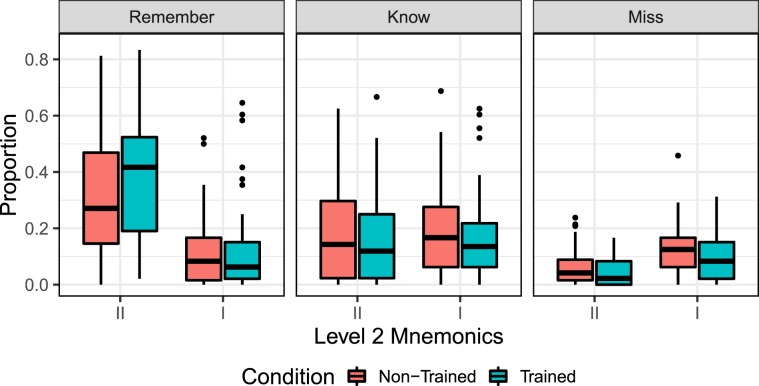
Descriptive statistics of AM psychometrics with respect to treatment conditions. Greater proportion of *R*-*II* response was found in the trained condition than non-trained condition - trained: 0.41 ± 0.23, non-trained: 0.32 ± 0.22, LH-test: 5.06, *p* = 4.1*e*-7. No statistical difference was observed between training conditions with respect to proportion of *R*-*I* (weak remember). An interaction existed with respect to the training_condition × event_detail: *z* = 2.15, *p* = 0.032. Combining *R*-*II* and *R*-*I*, the aggregated *R* rate is higher in the trained than non-trained condition with trained: 0.52 ± 0.22, non-trained: 0.42 ± 0.23, LH-test: *z* = 4.43, *p* = 9.4*e*-6. No statistical difference for “Know” categoriesbetween two conditions. *Miss* for both confidence levels (i.e., *M*-*II* and *M*-*I*) is lower under the trained than non-trained condition.

Among the mnemonic metrics, recollective AM is directly related to correct “Remember” responses (*R*), which consist of *R*-*II* (strong remember) and *R*-*I* (weak remember). Based on general linear hypothesis testing on training condition, training consistently led to higher rate of “Remember” (trained: 0.52 ± 0.22, non-trained: 0.42 ± 0.23, LH-test: *z* = 4.43, *p* = 9.4*e*−6). For level 2 mnemonic metrics, we found a higher proportion of *R*-*II* responses in the trained (0.41 ± 0.23) than non-trained condition (0.32 ± 0.22), LH-test: *z* = 5.06, *p* = 4.1*e*-7. Thus, the result indicated a boosting effect of training on event memory, i.e., retrieval practice enhanced the recollection by increasing the number of recollected events and inducing a higher level of recollected episodic details. Meanwhile, no difference in the *R*-*I* rate was found between the two conditions (trained: 0.10 ± 0.14, non-trained: 0.10 ± 0.12, LH-test: *z* = 0.35, *p* < 0.726). This denoted an interaction effect between training and amount of recollected event details (*z* = 2.15, *p* = 0.032), i.e., greater difference between *R*-*II* and *R*-*I* rates under the trained than the non-trained condition. This means that while training boosted the overall recollective memory, the boosting effect was more evident in increasing the amount of recollected episodic details. Meanwhile, a training effect was not observed with respect to the “Know” responses, indicating that training had little effect on gist-based familiarity. In addition, a training effect was observed with respect to *Miss* with high confidence (*M*-*II*: trained: 0.04 ± 0.05, non-trained: 0.06 ± 0.06, LH-test: *z* = −2.32, *p* = 0.02), and *Miss* with low confidence (*M*-*I*: trained: 0.14 ± 0.09, non-trained: 0.18 ± 0.11, LH-test: *z* = −3.40, *p* = 6.5*e*-4). A negative *z* value means lower ratio of such a memory type in the trained than non-trained condition, in agreement with the boosting effect of training. The result is shown in Fig. 2 (see Supplementary Table S1 for more details).

### Remember network overlap with the AM network

**Figure 3:**
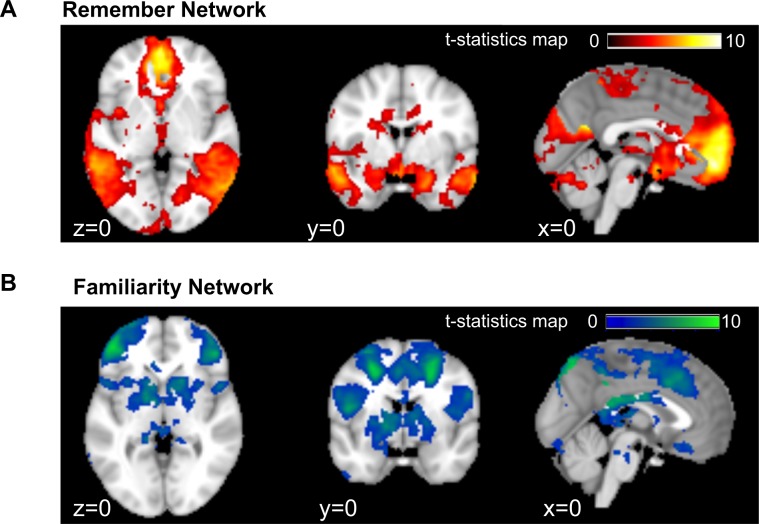
Brain regions associated with recollective memory vs. familiarity. (**A**) Widely distributed remember network that is selectively sensitive to recollection. Identified with events from both the trained and non-trained conditions using voxelwise nonparametric analysis by exclusively masking *R* > *K* contrast with the *K* > *Miss* + *CR* contrast. (**B**) Familiarity network identified using voxelwise nonparametric analysis by exclusively masking the *K* > *Miss* + *CR* contrast with *R* > *K* contrast.

For fMRI analysis, we first sought to identify brain regions that were selectively activated by recollection vs. familiarity. Regions were extracted by exclusively masking *R* > *K* contrast with the *K* > *Miss* + *CR* contrast^9^. In other words, the regions showed effects of recollection that did not also show familiarity effects. We identified widely distributed remember network (thereafter denoted as $\Re$) that coincided with the “AM network”^2,32^, as well as the “core recollection network”^23,24,41–44^. In particular, it spread across the medial prefrontal cortex(mPFC), medial temporal lobe (MTL) (including hippocampus, perirhinal cortex, parahippocampal gyrus, and retrosplenial cortex), parietal lobe (including bilateral angular gyrus, and precuneus), and occipital lobes (including cuneus, intracalcarine cortex, lingual gyrus, and occipital pole) (Fig. 3A). These regions also partially overlapped with elements of the default mode network^47–49^. It should be noted that the remember network was identified using data of both the trained and non-trained conditions, based on the consistent outcome when using data of either condition (refer to Supplementary Fig. S3).

We also identified brain regions that underpinned general event memory (i.e., familiarity), identified by exclusively masking the *K* > *Miss* + *CR* contrast with *R* > *K*^9^. The resultant familiarity network (denoted as *κ*) was largely dissociated with the remember network. In particular, it mainly located at lateral inferior frontal cortex, lateral middle frontal and precentral areas, middle cingulum, bilateral inferior parietal and supramarginal, and some areas of the precuneus, as shown in Fig. 3B.

### Amount of event details modulates activation of lateral temporal and parietal cortices

**Figure 4:**
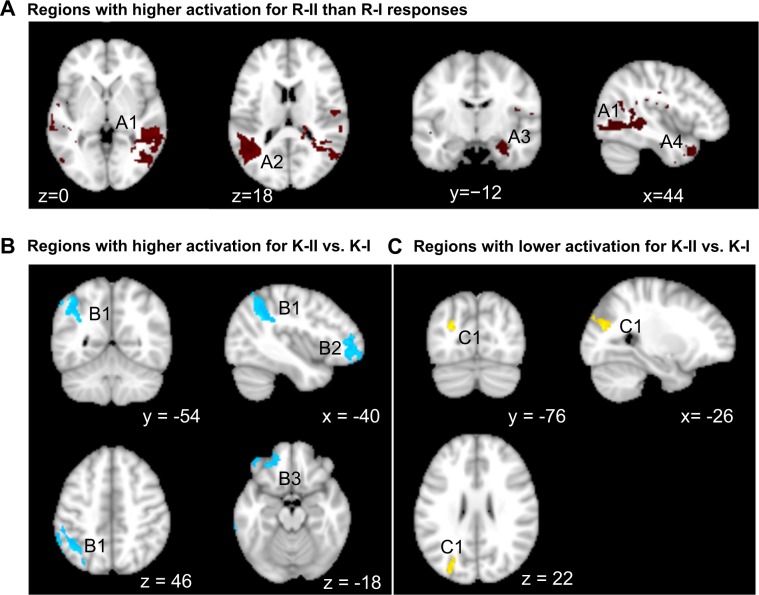
(**A**) Cortical areas within the remember network which activation is modulated by the recollected autobiographical event details. Identified using a voxelwise nonparametric second-level analysis based on the contrast of *R*-*II* > *R*-*I* at a threshold of *p* < 0.05 (cluster corrected), and inclusively masked by the remember network. (**B,C**) Cortical areas within the familiarity network which activation is modulated by the familiarity level. Identified using a voxelwise nonparametric second-level analysis based on the contrast of *K*-*II* > *K*-*I* and *K*-*I* > *K*-*II*, respectively, inclusively masked by the familiarity network. Refer to Table S2.

To test H1, a voxelwise nonparametric second-level analysis was carried out on the contrast *R*-*II* > *R*-*I* at a threshold of *p* < 0.05 (cluster corrected). To ensure that the effect was specific to regions associated with “remembered” events, the resultant *t*-statistics map was inclusively masked by the remember network $\Re$ ^9^. Differential activation for *R*-*II* vs. *R*-*I* was identified within right middle temporal gyrus, right superior temporal gyrus, right occipital cortex, left superior occipital cortex and angular gyrus, right hippocampus and parahippocampus, and right temporal pole (Fig. 4A). To rule out the possibility that the activation disparity were due to the content rather than the level of event details^9^, we examined if any brain regions were differentially activated by the reverse contrast, i.e., *R*-*I* > *R*-*II*. No voxels survived the reverse contrast.

We further conducted analysis within the familiarity network (*κ*) to verify if any regions were differentially activated by high vs. low familiarity. This was done by using similar second-level analysis on the contrast *K*-*II* > *K*-*I*; and further on the reverse contrast *K*-*I* > *K*-*II*, both at a threshold of *p* < 0.05 (cluster corrected). Three clusters were identified for the *K*-*II* > *K*-*I* contrast and one for the reverse contrast (Fig. 4B,C).

### Distributed training × event_detail interaction effect within remember network

To investigate the training effect, we carried out a voxelwise analysis to find brain regions for which the magnitude of the BOLD response presented an interaction between training condition and memory type (*R* vs. *K*) (H2.1), and an interaction between training condition and recollected event details (*R*-*II* vs. *R*-*I*) (H2.2). However, there were no surviving voxels following statistical correction (*p* < 0.05, cluster corrected) for both cases.

**Figure 5:**
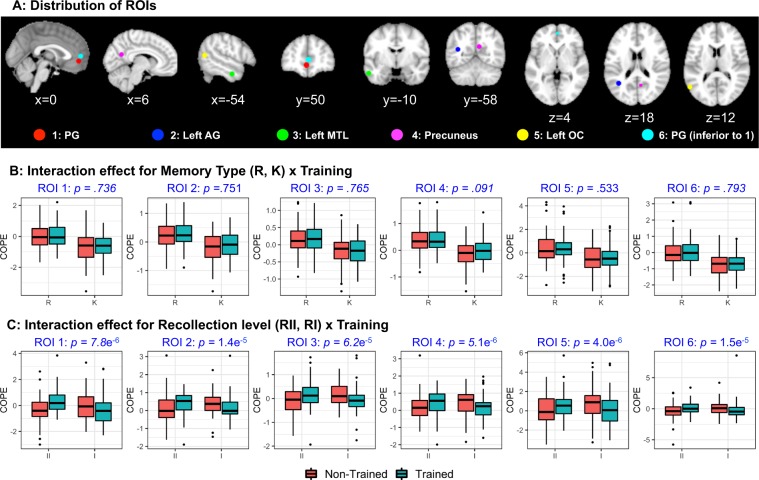
ROI analysis on the training_condition × event_detail interaction effect. (**A**) ROIs are defined by local maximas with 5 mm spherical kernels and corresponding boxplot showing COPEs extracted from each contrast. PG - paracingulate gyrus, AG - angular gyrus, MTL - medial temporal lobe, OC - occipital cortex. (**B**) No significant training_condition × memory_type (*R* vs. *K*) interaction effect could be found. (**C**) A significant training_condition × amount_of_event_detail (*R*-*II* vs. *R*-*I*) interaction effect was found within all ROIs. Increased *R*-*II* response amplitude relative to *R*-*I* was observed in the trained relative to non-trained condition.

To account for the lack of sensitivity of voxelwise analysis, ROIs related to recollective memory were selected within the remember network ($\Re$) based on local maximas^50^. Six ROIs were defined by a spherical kernel of 5 mm radius from the local maximas of *t*-statistics within $\Re$, including (1) paracingulate gyrus, (2) left angular gyrus, (3) left middle temporal gyrus, (4) precuneus, (5) left inferior occipital cortex, and (6) paracingulate gyrus (inferior to ROI 1), as illustrated in Fig. 5A. The mean contrast of parameter estimates (COPE) for *R*_*T*_, *R*_*N*_, *K*_*T*_, and *K*_*N*_, denoting the fitted amplitude of the BOLD response at the corresponding events, were extracted for the six ROIs. The effect of training_condition × memory_type (*R* vs. *K*) was examined, but an interaction effect was not established (Fig. 5B). In contrast, a significant interaction effect (*p* < 0.05, corrected for multiple comparisons) were established regarding the mean COPE for *R*-*II*_*T*_, *R*-*I*_*T*_, *R*-*II*_*N*_, and *R*-*I*_*N*_ in all six ROIs (Fig. 5C). It denoted increased *R*-*II* response amplitude relative to *R*-*I* in trained relative to non-trained condition, indicating that training led to higher cortical differences between high vs. low levels of episodic details. This pattern was evident in all ROIs, which spread across the remember network. ROI analysis on the familiarity network is given in Supplementary.

## Discussion

Retrieval practice enhanced recollection of autobiographical events as seen from the greater proportion of successfully recalled personal events under trained than non-trained condition. This was consistent with existing evidences in both the laboratory^37,51^ and the real-world environments^32,52,53^. We also observed a main effect of training on increasing the proportion of events with more episodic details. In addition, an interaction effect of event_detail × training_condition indicated that the training was particularly useful in maintaining rich episodic details. This coincides with the observation that repeated retrieval facilitated stronger visual imagery, which is one of the characteristics of episodic richness^53^. Assuming that retrieval practice and reactivation facilitate similar enhancement effect on AM, and that reactivation quality is generally reduced in older adults^32^, such an enhancement effect might be reduced in older adults. Our results showed that despite the potential reduction of enhancement effect in this population, it still persisted so that retrieval-based training was effective in helping older subjects maintain better recollective memory. In fact, a related comparative study involving young and older adults showed an age-invariant effect of ESI-based training on memory^15,54^. Our results thus supported the view that older adults could benefit from cognitive training that selectively oriented toward episodic construction.

More importantly, we examined the neural basis of autobiographical event memory and the retrieval-mediated boosting effect. First, brain regions that were selectively sensitive to the recollection of event details were found within mPFC, MTL, bilateral angular gyrus, precuneus, cuneus, and occipital pole. These regions overlapped with the AM network that have been identified in various conditions (notably from young adults)^4,5,48,55,56^. This network overlapped with the remember network and the “core recollection network”^41–43,57^, which indicated a common basis of recollective memory irrespective of its personal relevance. The separated regions of the remember network and the familiarity network extended past findings on the dissociation of neural correlates of specific and general AM by showing its presence in the real-world settings. Three studies have examined this specific issue in the real-world. First, a few clusters were identified to track recollective memory within the mPFC and MTL (including right anterior parahippocampal gyrus, and right posterior parahippocampal gyrus)^28^. In comparison, this research found widely distributed network across the brain. Such disparity may be attributed to a few methodological and operational differences, notably, sample size (42 vs. 15 subjects; 192 vs. 80 trials/subject), age of subjects (old vs. young), method of analysis (whole-brain analysis vs. *a prior*i interest in the MTL region), and the duration of lifelog data collection (30 vs. 2 days^28^). The last issue was especially important because shorter data collection time meant densely sampled events (e.g., 40 events/day^28^), which may lead to increased memory interference in the recall test. In comparison, this research collected the lifelog data over 30 days with about 6 events per day. This would significantly alleviate memory interference caused by similar memory cues. Recently, similar conditions were adopted in an experiment (though with young participants), and a remember network was identified within medial frontal, bilateral anterior insula, left angular and supramarginal gyri, retrosplenial cortex/posterior cingulate cortex, and bilateral regions of the hippocampus and parahippocampal cortex^27^. These results were compatible with ours, so that the remember network seemed to be stable among different age groups. Finally, anterior MTL was found to be sensitive to the episodic AM based on ROI analysis using a subset of data collected from two real-world experiments^7^. Exploratory voxel-level analysis showed that recollection was associated with extended regions from bilateral entorhinal/perirhinal cortex to the hippocampal head and amygdala, and ventral regions of angular gyrus in the parietal cortex^7^, which partially overlapped with the remember network identified in this study.

Neural correlates of AM at refined levels of recollection was further manifested by the existence of brain regions that showed disparate activation for event memories with rich details relative to those with scant details. Such a disparity was found in right hippocampal and parahippocampal areas, right middle/superior temporal gyrus and temporal pole, right occipital cortex, left angular gyrus and superior occipital cortex. There was limited and inconsistent evidence on the neural substrates that tracked the amount of episodic details recollected in real-world settings. For instance, no disparity was observed given the absence of voxel clusters (cluster extent >45) that were differentially activated for strong (vs. weak) remember, whereas such an effect was identified within left angular gyrus and bilateral vmPFC when relaxing the cluster extent requirement^27^. We also found differentiated activation due to amount of event details in left angular gyrus, in agreement with the above evidence. However, we did not observe such an effect in vmPFC. This can be explained by the age-related change in the role of PFC in AM retrieval. Specifically, the top-down influence PFC components on hippocampus which was considered to modulate episodic richness was reduced in older adults as compared to young adults^30^. In other words, aging impacts the strategic control (mediated by members of PFC) during the AM retrieval. The lack of involvement of vmPFC in this process as observed in this research corroborated such an age-related change. In lined with this observation, the dorsal anterior cingulate cortex - a region considered to facilitate executive control during the AM retrieval - showed reduced involvement in the recall of episodic AM among older adults relative to young adults^12^. It was reported that differential activation for strong vs. weak recollection was observed within left posterior parahippocampal gyrus and left hippocampus^28^. This was different from the our result where such an effect was observed in right hippocampus/parahippocampus. The disparity could be attributed to an age-related shift of the hippocampal contribution to episodic memory retrieval^30^. While left hippocampus was considered to track the level of details during AM retrieval^4^. For older adults, right hippocampus may become more sensitive to the variability of episodic detail of the spatial and emotional context^30,58^.

Beyond the specific context of autobiographical event memory, there are many works that explored the neural mechanisms of similar alternative cognitive processes. Notably, the usage of photos as visual cues has a bearing on scene imagery and memory^59,60^. Indeed, many of the stimuli used in this experiment were scenes, with diverse, rich semantic contents, such as human faces and body parts, and objects. This explains the commonality of the remember network identified in this study with the scene-processing network^61^. Specifically, components of the remember network included many elements of the ventral visual pathway that are responsible for scene processing, including parts of the occipital lobes, parietal lobes, parahippocampal gyrus, and retrosplenial cortex^60–62^. The involvement of areas extending from the vmPFC to hippocampal areas corroborates existing findings that synchronized neural activity between these two regions supported mental generation of scene imagery^59^. Some elements of the scene network were co-located with the regions showing differentiated activation due to varying levels of recollected event details. These observations were in agreement with the theory of event memory that postulates the essential role of scene construction in event memory retrieval^6,63^, and recollective experience^64^. In a similar vein, this research identified cortical areas that were sensitive to the level of episodic details, which shared commonality with regions that tracked vividness of recollection^35,39^. In addition, given the strong social context of autobiographical event memory, the networks identified in this experiment consistently involved the angular gyrus, which has been considered to represent social context under the hood of the theory of mind^65–67^. This can be explained by our experiment protocol, i.e., the cued-recall task, by necessity, required the subjects to make self-projection into the presented scenes, as long as it incurred a sense of reliving of the respective experiences.

Finally, we examined the effect of retrieval-based training on the recollective quality of AM. Although such an effect was not established based on voxelwise analysis, we found a consistent training effect in six ROIs. Particularly, training facilitated greater difference in neural responses between strong and weak recollective memory, i.e., (*R*-*II*_*T*_ − *R*-*I*_*T*_) > (*R*-*II*_*N*_ − *R*-*I*_*N*_), whereas a statistically significant difference was not observed for (*R*_*T*_ − *K*_*T*_) > (*R*_*N*_ − *K*_*N*_) contrast. This result is surprising at first glance because both *R* − *K* contrast and *R*-*II* − *R*-*I* contrast denote variations of episodic details. Specifically, the former reflects the presence/absence of episodic details, and the latter reflects different levels of episodic details, given its presence. Thus, they should show similar training effects, if any, according to the constructive episodic simulation hypothesis^3,15^. The ostensible inconsistency can be explained by a dilution effect when combining the two mnemonic categories. In essence, retrieval practice may be effective in modulating the retrieval orientation towards episodic details only for events recalled with relatively richer episodic detail, and not so much for events with lesser episodic detail. The interaction effect in (*R*-*II*_*T*_ − *R*-*I*_*T*_) > (*R*-*II*_*N*_ − *R*-*I*_*N*_) strongly support such an observation. When combining event memory with both high and low levels of episodic details, i.e., *R* vs. *K*, the neural response in the *R*-*II* category might have been diluted by the *R*-*I* category, causing the absence of the training effect at the more coarse level. This coincided with a similar interaction effect observed in the behavioral metrics, namely, relatively higher difference between the proportion of *R*-*II* and *R*-*I* under trained than non-trained condition. Both results showed a more evident boosting effect of training on memory with rich event details. Finally, the absence of an interaction effect involving different familiarity levels and training condition (*K*-*II*_*T*_ − *K*-*I*_*T*_) > (*K*-*II*_*N*_ − *K*-*I*_*N*_) was expected because by definition, familiarity levels are independent of episodic details. Therefore, their contrast was not affected by training.

There are several neurocognitive accounts of the ROI-based interaction effect in the recollection of autobiographical event details. First, according to the ESI effect^3^, training may facilitate greater activation in members of the core recollection network, such as, bilateral anterior hippocampus, right inferior parietal lobule, and right ventral precuneus. In lined with the above finding, retrieval practice as adopted in this experiment did facilitate “deeper” involvement of multiple core network regions (i.e., the ROIs) in the recollective memory process. Although the ROIs were not strictly co-located with the regions reported in ESI study, both belonged to the core recollection network that supports memory of episodic details. A cluster-based comparative analysis was not conducted though, since neither analysis survived more conservative statistical control. Methodologically, our work differed from the ESI study as the latter only studied the effect of training based on the performance disparity in two distinctive types of tasks - one relying on episodic retrieval, and the other relying on semantic object comparison. These categories were not equivalent to the *R*-*II* vs. *R*-*I* categories which differed in the amount of episodic details. Therefore, our findings extended the ESI by showing the training effect on the refined levels of recollective AM. Moreover, we showed that the training effect was present in older adults on a similar set of neural substrates, which suggested that the neural basis of the training effect may be similar across age groups.

Second, based on the principles of reactivation-based memory reconsolidation^34,36^, retrieval of an episodic memory places it into a labile state so that the memory traces are updated. The retrieval practice allows the cortical representations of events to be reactivated or reinstated, which not only consolidates and stabilizes the original memory traces^8,68^, but also leads to the transformation/elaboration of them owing to the presence of new information (in this case, lifelog photos)^34,36,37^. Assuming that the reported recollected event details reflects the strength of the original memory traces, the result suggested that the above process might be particularly effective for memories with stronger traces (perhaps due to more effective initial encoding). That is, the visual cues might have strengthened the memory by allowing it to bind with rich spatial or emotional context that otherwise became inaccessible. Meanwhile, memories with weaker memory traces might not have benefited as much from the above process. In comparison, for non-trained events, the event episodes (irrespective of the strength of the memory traces) were not reactivated and underwent normal decaying, leading to reduced distinctions of cortical activation between strong vs. weak memory. From the neurocognitive perspective, reactivation-induced updating has been considered to be associated with the recruitment of left posterior parahippocampal, retrosplenial, and posterior inferior parietal cortices during AM retrieval^34^. These regions were closely co-located with the ROIs that exhibited the training effect in this research. As such, our results not only affirmed the neural basis of reactivation-induced updating, but also extended it by showing the effect among older adults. In essence, despite an age-related change of AM retrieval with respect to recollective memory^30^, the neural basis of intervention, be it reactivation^34^ or retrieval practice, may be shared to a certain extent among different age groups.

The neural correlates of recollective event memory and the training effect reported here should be interpreted with caution considering methodological and operational limitations of the experiment. First, we recruited only older adults in this experiment. Therefore, the findings may not be directly applicable to other age groups, although we have identified notable similarity between our results and previous findings from general populations. Second, it is difficult to attribute the aforementioned accounts to specific neural processes in the ROIs. In order to examine the neural process of reactivation-based reconsolidation or retrieval-induced forgetting, one needs to track the brain activation patterns during encoding and training, and associate them with those collected during testing, as well as the memory outcome^34,69^, e.g., using representational similarity analysis^37,51,70,71^. However, due to operational constraints in the real-world, this study did not record neural process during encoding and training, making it impossible to check the reinstatement effect. Nevertheless, an interaction effect was observed within all 6 ROIs, which spread across the remember network. As such, it was unlikely that such an effect had happened out of chance. Third, the comparisons with results from the reactivation-based training^34^ and ESI-based training^3^, particularly concerning the possible common neural basis of the training effect across age groups, was not backed with direct neural evidence on a common protocol. To get more convincing evidence on age-related effects, future work can be carried out to replicate the present protocol with young adults, and compare the outcome with the present work.

In summary, the current study showed that retrieval practice of real-life events was effective in enhancing event recall among older adults. Widely distributed remember network as separated from the familiarity network supported the view of dissociable process of recollective memory and familiarity. This extended previous findings which only observed such a process in the laboratory settings. The lateral temporal and parietal regions were differentially activated for AM with higher (vs. lower) episodic details. The lack of involvement of vmPFC regions, as was different from previous studies with young adults, showed a possible age-related change in the role of vmPFC in modulating episodic recall. The training_condition × event_detail interaction effect observed in the ROIs of the remember network showed the complicated role of retrieval practice on autobiographical event memory. This offered new insights that retrieval practice enhanced episodic memory where the effect was modulated by the strength of memory traces; and that the neural mechanism of intervention might be invariant to ages. Finally, the findings have important practical relevance in the design of cognitive intervention programs, e.g., to selectively strengthen episodic memories by choosing photo cues that facilitate greater memory change^72^ and the evaluation of the efficacy of such intervention programs by establishing biomarkers around the regions that track the episodic richness of autobiographical memory.

## Supplementary information

Supplementary information.

Supplementary Dataset 1.

## Data Availability

The data generated/analysed during the study are available from the corresponding author on reasonable request. Main fMRI data are included in the Supplementary Information files.
