# Supplementary material for: Neural correlates of retrieval-based enhancement of autobiographical memory in older adults
Source: Sci Rep. 2020 Jan 29;10:1447. doi: 10.1038/s41598-020-58076-6 (PMC6989450; doi:10.1038/s41598-020-58076-6)
Supplement: Supplementary file 1 — Supplementary information. [file 41598_2020_58076_MOESM1_ESM.pdf]

Neural correlates of retrieval-based enhancement of autobiographical memory in older adults

Supplementary Information

Qianli Xu[1†*], Jiayi Zhang[2†],Joanes Grandjean[2],Cheston Tan[1,3], Vigneshwaran Subbaraju[1,2,3], Liyuan Li[1],Kuan Jin Lee[2], Po-Jang Hsieh[4], Joo-Hwee Lim[2]

1. Institute for Infocomm Research, Agency for Science, Technology and Research (A*STAR), Singapore
2. Singapore BioImaging Consortium, Agency for Science, Technology and Research (A*STAR), Singapore
3. A*STAR Human-Centric Artificial Intelligence Programme, Singapore
4. Department of Psychology, National Taiwan University, Taiwan

† these authors contributed equally

* Corresponding author

Cued-recall for Memory Training

In a trained cycle (denoted as 'T'), subjects were instructed to wear the camera for 6-8 hours per day. After 3 or 4 days, subjects visited the experiment site for a cued-recall training process. During this visit, lifelog data was downloaded from subjects' device, and the experimenter manually selected "eventful" photos from the current cycle. On average, 10 photos were selected per day as visual cues for the training, which was considered as a balanced sampling strategy (Rissman et al. 2016; St Jacques et al. 2011). Next, subjects were shown the selected photos and were asked to recall events related to them. They were prompted to describe the events by

providing episodic details, such as time, place, people, and context, instead of describing the visual content of the photo only. This was intended to stimulate an effortful recall rather than a passive re-study (Wing et al. 2013) and facilitate a specific retrieval orientation within the subjects (Madore et al. 2016). In a non-trained cycle (denoted as 'N'), subjects collected lifelog data, which were downloaded during their visits; but they did not participate in the cued-recall training. A trained session usually lasted about 1 hour and a non-trained session lasted about 30 minutes. The intervention time was different under two conditions, but we considered such a difference would not affect subsequent memory performance because the study was conducted in the real-world and an additional 30-minute non-task related waiting time under the non-trained condition was not much different from other personal activities. The experiment had two phases, which differed with respect to the order of T-N cycles. Subjects were randomly divided into two groups with different combinations of T-N cycles. Both arrangements were intended to account for the possible differences in study-recall interval, within and between subjects.

In-scanner Autobiographical Recall Test

In the test session, subjects were informed that they would be shown photos that were captured either by themselves or by others. Subjects were instructed to answer two questions after seeing a photo. The first question was about the type of memory of the cued event. There were three possible responses: *Remember*, *Know*, and *New*, in accordance with the 'Remember-Know' paradigm (Milton et al. 2011; Rajaram 1993; Rotello et al. 2004). "*Remember*" is defined as the recollection of specific details and context of an event, which incurs a feeling of re-experiencing the moments. "*Know*" refers to a general feeling of familiarity without recalling event-specific

details. "*New"* is something not from one's own life. The second question further inquired the level of the respective memory. For a "*Remembered*" event, subjects could choose from one of four levels of recollected detail, where "1" indicates a few details, "4" indicates many details, and "2" and "3" are intermediate levels. For a "*Known*" event they would choose from four levels of familiarity, where "1" means low familiarity, "4" means high familiarity, and "2" and "3" are intermediate levels. For a "*New*" event, they would report their levels of confidence, namely "*Certainly not mine*" or "*Perhaps not mine*". The second question was intended to investigate subjects' judgment at refined granularity, similar to the confidence of primary judgment (Leiker and Johnson 2015). In addition, "*Remember*" and "*Know*" adopted more refined granularity (4 levels) than "*New*" (2 levels), because they were more relevant to recollective AM, i.e. the main focus of the study.

The in-scanner memory test had a total of 144 trials that were divided into 8 runs, each consisting of 18 trials with one stimulus in each trial. Among the 18 stimuli, 6 were drawn from the trained condition, 6 from the non-trained condition, and 6 were lure images. Each run started with a 10 s blank period during which a white fixation cross was displayed in the grey background. Next, a trial began with a jitter of 4 ± 2 s, followed by a stimulus shown for 8 s in a grey background. Next, three answer boxes were displayed on the screen, namely "*Remember*", "*Know*" and "*New*". "*Remember*" and "*Know*" were always displayed on the same side, and was opposite to "*New*" (Figure S1). The corresponding item was highlighted in green once chosen. Three seconds after the onset of Question 1, the answer boxes of Question 2 was shown. If the previous judgment was "*Remember*", four boxes (1, 2, 3, and 4) were shown under the title "*Detail*". If the previous judgment was "*Know*", four boxes (1, 2, 3, and 4) were shown under the

title “*Familiarity*”. If the previous judgment was “*New*”, two boxes were displayed: “*Certainly not mine*” and “*Perhaps not mine*”. If the subject did not respond in Question 1, a blank screen was shown for 3 s as a placeholder. Each run ended with a 10 s blank period. Two test sessions (also called scans) were arranged for each subject. Responses were recorded using two 4-button boxes. The button box in the left/right hand corresponded to the left/right side of the display. The fingers were mapped vertically to the stack of answer boxes on the screen, i.e. the index finger triggered the topmost item, followed by the middle, ring, and pinky fingers in that order. To account for cognitive process related to finger motion, four configurations were designed according to the lateral and vertical layout of the answer boxes and the layouts were assigned pseudorandomly and counter-balanced for different subjects (Figure S1). The stimuli presentation and the timing of response were controlled by the Psychtoolbox (Brainard 1997) in Matlab (Mathworks, Natick, MA, USA). The visual information was projected onto a screen, which was viewed through a mirror mounted on a helmet.

Selection of Photo Stimuli for Recall Test

The above in-scanner recall test used photo stimuli that were selected from the lifelog photos. They were selected manually by experimenters who were blind to the training conditions. Photos were chosen if they were of personal relevance to the subject, applying the same criteria as in the selection of photos for cued-recall during training. Images were excluded if they were of poor quality (e.g. due to excessive movement), or showed the subject’s own body part (e.g. hands and arms). Critically, an image was excluded if it had been used in the cued-recall during training. This was intended to reduce the effect of alternative cognitive processes such as photo

perception, which may co-vary with the main experiment factor, i.e. training. On average, 10 photos were selected from each day when data collection took place. Furthermore, the following procedure was adopted to avoid biased sampling in favor of certain content for specific conditions. Photos were encrypted as three conditions, i.e. trained, non-trained, and lure, with 70~80 photos in each condition. Next, all the photos were annotated according to whether it (1) had a human face (2) had a human body (with or without face) (3) was taken indoor or outdoor. In addition, (4) an image memorability score was computed which indicated the visual saliency of the image (Isola et al. 2014; Bainbridge and Rissman 2018). Finally, 54 photos were drawn pseudorandomly from each of the conditions so that they were equivalent with respect to the above four criteria. Among the 162 (54×3) stimuli, 18 were randomly selected and used for practice and the rest 144 were used for the actual test.

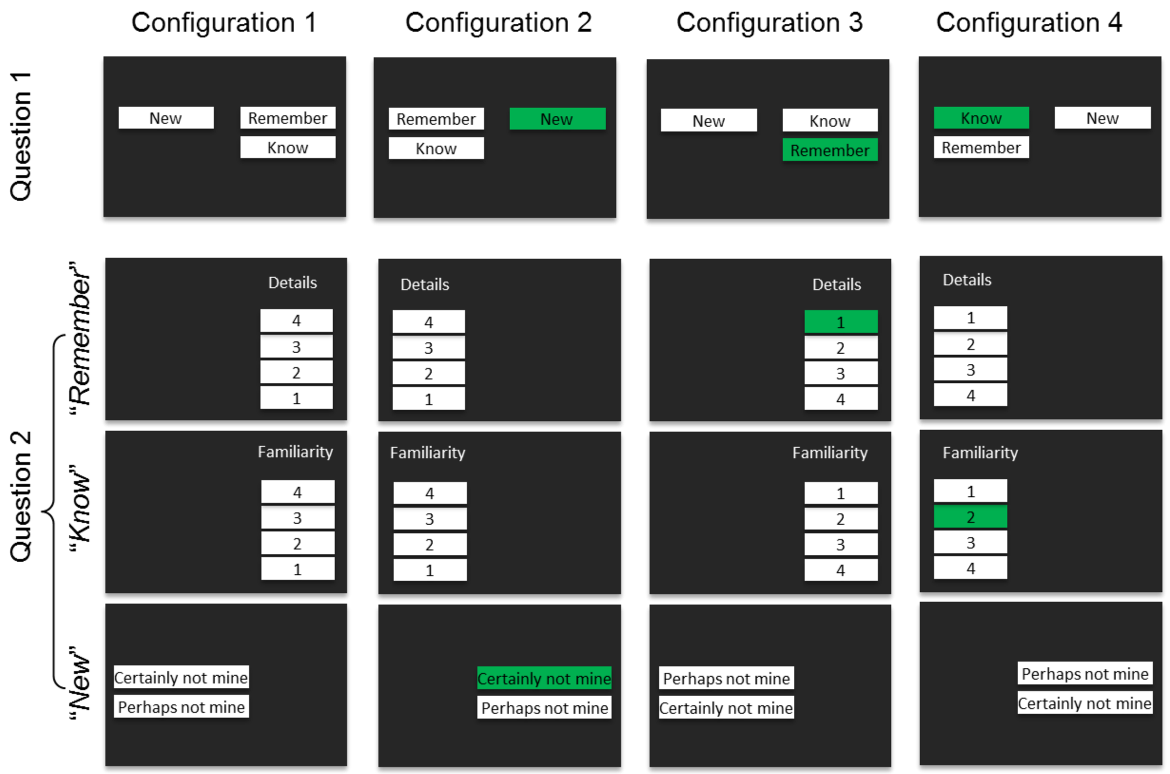

Figure S1. Four configurations of response display during in-scanner memory test. Each scan uses two configurations, which are laterally mirrored, i.e. (Configurations 1 and 2, or Configurations 3 and 4). Note that depending on a subject's answer in Q1, only one visual prompt (corresponding to "Remember", "Know", and "New, respectively) appears for Q2. Each configuration is used in 4 consecutive runs, before which a short training is performed. Configurations 1 and 2 adopted similar vertical layout and different (mirrored) lateral layout. The lateral layout of configurations 3 and 4 were identical, whereas they vertical layout was different from configurations 1 and 2. Subjects were randomly assigned to two configurations with similar vertical layout but different lateral layout - one configuration used for the first half of the scan (4 consecutive runs), and the other used for the second half of the scan. The assignment was pseudorandom and counter-balanced for different subjects.

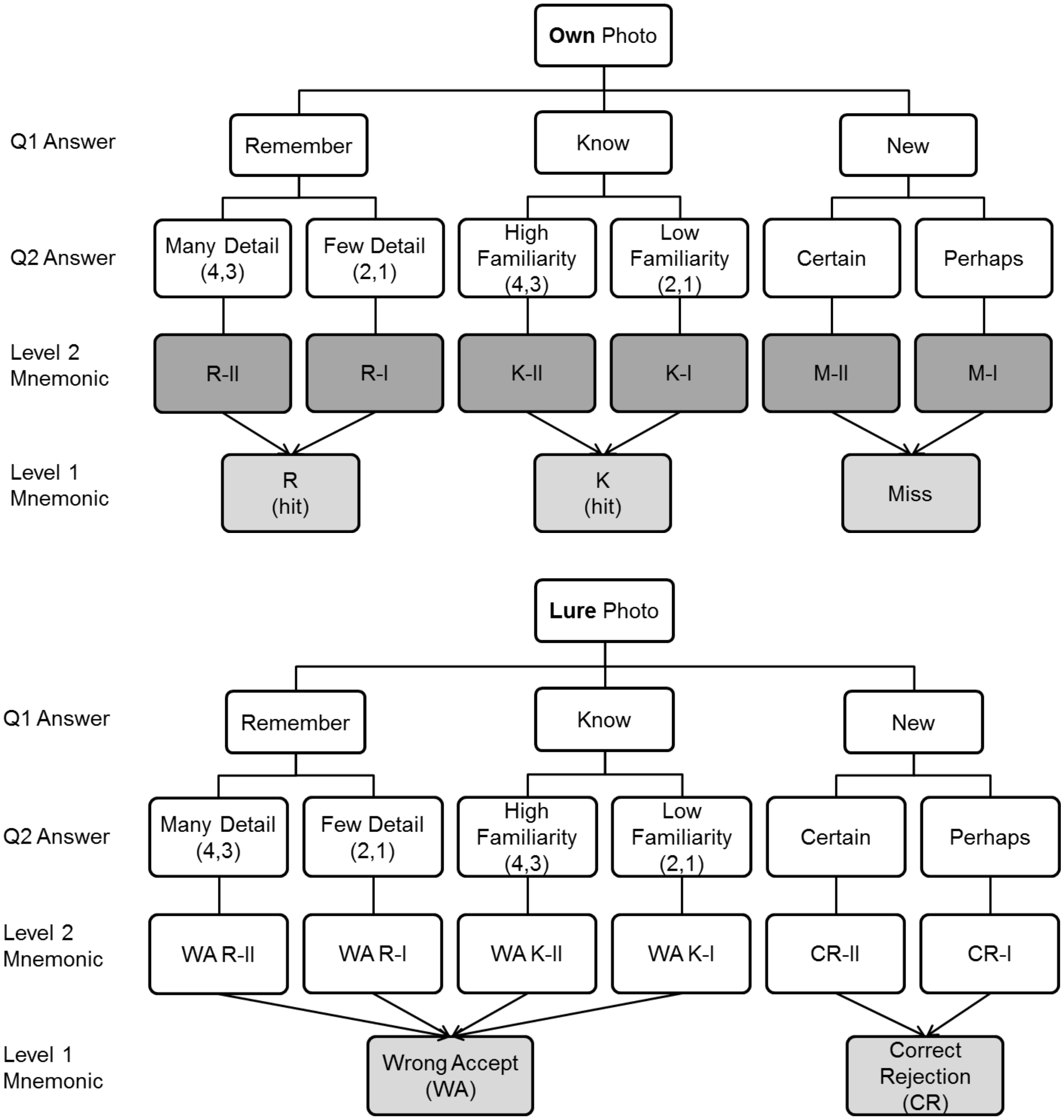

Figure S2. Cued-recall test answers and mnemonic types. Boxes with light-shade are the mnemonic types used for first-level analysis. Boxes with dark-shade are mnemonic types for 2nd level analysis.

A Priori Exclusion Criteria

The behavioral (i.e. the subjective report of memory in the testing) and fMRI data was filtered according a few criteria to exclude abnormal results caused by subject non-compliance, e.g. inattention, excessive guess work, misunderstanding of tasks, etc.

1. Absence of answers in 3 or more (out of 18) stimuli prompts in a run => exclude run.
2. Hit rate (including remember and know responses) < 50% and/or CR (correct rejection) <50% in a run => exclude run.
3. Hit rate <60% and/or CR(correct rejection) <60% in a scan => exclude scan.
4. Absence of 'Remember' or 'Know' answers in a scan => exclude scan.
5. Absence of weak remember (Remember detail levels 1 and 2) or strong remember (Remember detail levels 3 and 4) in a scan => exclude scan.
6. Absence of low familiarity (familiarity levels 1 and 2) or high familiarity (familiarity detail levels 3 and 4) in a scan => exclude scan.

In addition to the above criteria, fMRI analysis further excluded scans with excessive head motion.

7. Maximal motion in scan was greater than 5mm => exclude scan.
8. 2 scans excluded in (3~7) => exclude subject.

In total, 437 and 237 runs of data were included for the first-level and second-level analysis respectively. The sample size was 37 because both scans of one subject were excluded.

Identify Remember Regions under Two Training Conditions

In the main text, remember network was identified using data of both the trained and non-trained conditions. Considering that training may affect the neural processes, it was possible that the network was different under these two conditions. We examined this issue by establishing two maps of remember network using data samples from either trained or non-trained condition, respectively. A group-wise nonparametric two sample *t*-test revealed that for the regressor R > K there is no significant difference between the trained and non-trained condition ($p < 0.05$, cluster corrected) (Figure S3). Therefore, events from both conditions were combined in the estimation of the remember network.

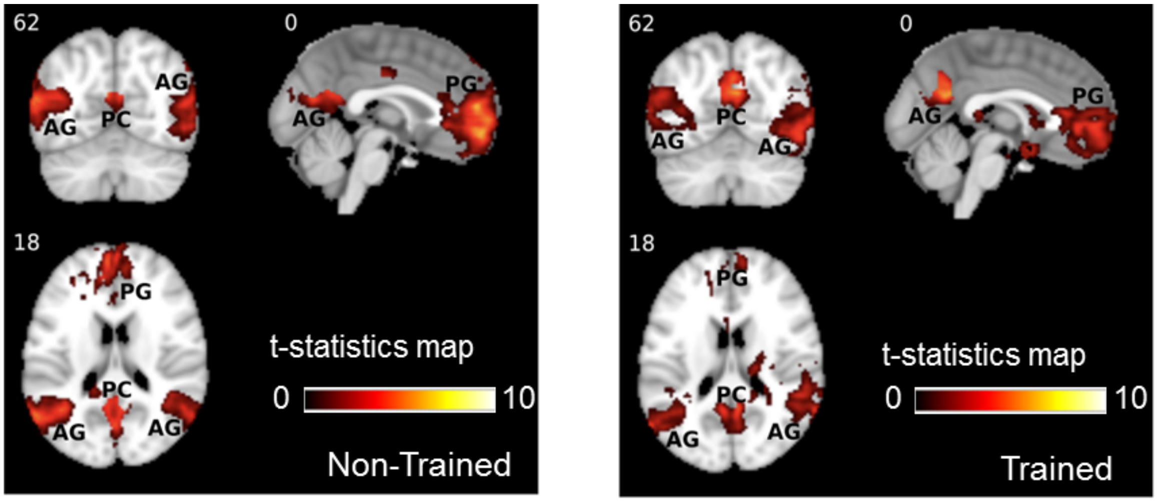

Figure S3. Remember network identified exclusively from either trained or non-trained condition events. This regions were identified using voxelwise nonparametric analysis by exclusively masking the R > K contrast with K > Miss + CR contrast.

Table S1. Descriptive statistics of all mnemonic types that show the recall performance. Training led to higher episodic details as is indicated by a higher ratio of R-II response. It also resulted in lower Miss rates, i.e. own photos recognized as “*New*”.

**Mnemonic Type**	**Non-Trained**		**Trained**		**LH-test *z***	***p* value**
	Mean	Std dev.	Mean	Std dev.		
R-II: Strong Remember	0.32	0.22	0.41	0.23	5.06	4.1e-7***
R-I: Weak Remember	0.10	0.12	0.11	0.14	0.35	0.726
K-II: High Familiarity	0.19	0.19	0.17	0.17	-1.19	0.236
K-I: Low Familiarity	0.20	0.16	0.17	0.14	-1.66	0.096
M-II: Miss Certain	0.18	0.11	0.14	0.09	-2.32	0.02*
M-I: Miss Perhaps	0.06	0.06	0.04	0.05	-3.40	6.5e-4***

Table S2. Brain regions showing selective sensitivity to the memory level. Coordinates are in MNI space. Significance level $p < 0.05$ (cluster corrected).

	Region	x	y	z	Size (voxels)	Peak *t*
**R-II > R-I (in the remember network)**						
1	Right middle temporal gyrus, right superior temporal gyrus, right occipital gyrus	44	-38	2	2362	4.00
2	Left superior occipital cortex, left AG	-48	-62	20	1036	4.36
3	Right hippocampus, right parahippocampus	24	-18	-20	482	4.15
4	Right temporal pole	44	20	-32	160	4.23
**K-II > K-I (in the familiarity network)**						
1	Supramarginal cortex	-56	-42	34	1092	5.03
2	Left middle frontal cortex	-36	46	4	887	4.77
3	Middle orbitofrontal cortex	-26	40	-18	105	4.01
**K-I > K-II (in the familiarity network)**						
1	Left middle occipital cortex	-30	-78	22	107	3.14

Combining Data of Two Scans

Since the experiment had two scans in two phases, we first examined whether the behavioral performance was affected by the scan order. We conducted general linear hypothesis testing on training condition (trained vs. non-trained) × scan order (scan 1 vs. scan 2), on all the mnemonic categories. As shown in Table S3. There was an absence of interaction effect in most except two items (Miss: LH-test, z=2.31, p=0.021; Miss-Certain: LH-test, z=2.02, p=0.043). Given the

dominant absence of statistical significance on all other mnemonic categories, we accept the null hypothesis regarding the interaction effect. Moreover, we did not find a main effect of scan order with respect to all the mnemonic categories, as seen from the last two columns of Table S3. Based on these results, we combined the behavioral data of two scans.

Table S3. Test of interaction effect (training condition × scan order) of all mnemonic types. Test of main effect of scan order on mnemonic types.

**Mnemonic Type**	**Training × Scan order Interaction**		**Scan Order Main Effect**	
	**LH-test *z***	***p* value**	**LH-test *z***	***p* value**
Remember	-1.18	0.240	0.02	0.981
Strong Remember (R-II)	-1.69	0.091	0.43	0.665
Weak Remember (R-I)	0.36	0.721	-0.60	0.547
Know	0.09	0.930	-0.30	0.763
High Familiarity (K-II)	-0.26	0.795	-0.95	0.341
Low Familiarity (K-II)	0.24	0.809	0.55	0.584
Miss	2.31	0.021^*	-0.12	0.907
Miss Certain (M-II)	2.02	0.043^*	0.40	0.691
Miss Perhaps (M-II)	1.11	0.266	-0.36	0.717

ROI Analysis of Training Effect in the Familiarity Network

We examined if training influenced level-2 mnemonics on "*Know*" responses. To do so, we checked for potential interaction effect in a voxelwise second-level analysis in the familiarity network ($\mathcal{K}$) using regressors K-II$_T$, K-I$_T$, K-II$_N$, and K-I$_N$. No interaction effect were found.

Further analysis within ROIs in $\mathcal{K}$ (which were identified using a similar method as for $\mathfrak{R}$ ROIs), did not show an interaction effect either (Figure S4).

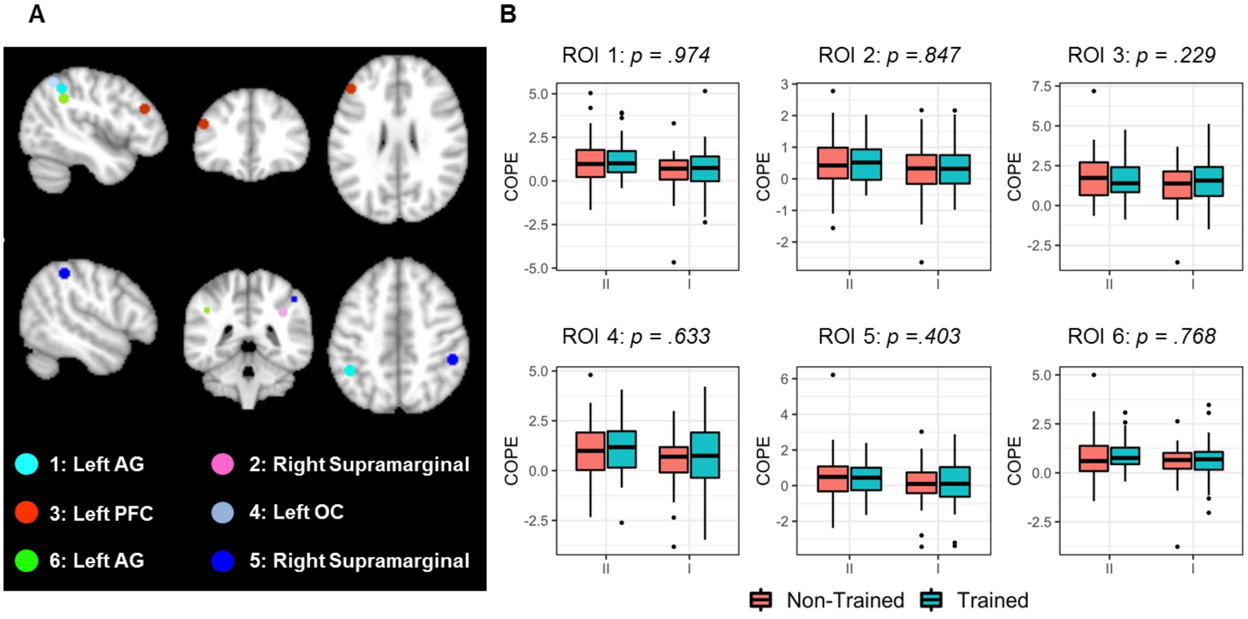

Figure S4. ROI analysis on the training condition × familiarity level interaction effect

A. ROIs as defined by local maximas with 5mm spherical kernels and corresponding boxplot showing COPEs extracted from each contrast. AG - angular gyrus, PFC - prefrontal cortex, OC - occipital cortex. **B**. An interaction effect was not established for (training condition) × (familiarity level: K-I vs. K-II) within all ROIs.

References

Bainbridge, W.A. and Rissman, J. 2018. Dissociating neural markers of stimulus memorability and subjective recognition during episodic retrieval. *Scientific reports* 8(1), p. 8679.

Brainard, D.H. 1997. The Psychophysics Toolbox. *Spatial Vision* 10(4), pp. 433–436.

Isola, P., Jianxiong Xiao, Parikh, D., Torralba, A. and Oliva, A. 2014. What makes a photograph memorable? *IEEE transactions on pattern analysis and machine intelligence* 36(7), pp. 1469–1482.

Leiker, E.K. and Johnson, J.D. 2015. Pattern reactivation co-varies with activity in the core recollection network during source memory. *Neuropsychologia* 75, pp. 88–98.

Madore, K.P., Szpunar, K.K., Addis, D.R. and Schacter, D.L. 2016. Episodic specificity induction impacts activity in a core brain network during construction of imagined future experiences. *Proceedings of the National Academy of Sciences of the United States of America* 113(38), pp. 10696–10701.

Milton, F., Muhlert, N., Butler, C.R., Benattayallah, A. and Zeman, A.Z. 2011. The neural correlates of everyday recognition memory. *Brain and Cognition* 76(3), pp. 369–381.

Rajaram, S. 1993. Remembering and knowing: two means of access to the personal past. *Memory & Cognition* 21(1), pp. 89–102.

Rissman, J., Chow, T.E., Reggente, N. and Wagner, A.D. 2016. Decoding fMRI signatures of real-world autobiographical memory retrieval. *Journal of Cognitive Neuroscience* 28(4), pp. 604–620.

Rotello, C.M., Macmillan, N.A. and Reeder, J.A. 2004. Sum-difference theory of remembering and knowing: a two-dimensional signal-detection model. *Psychological Review* 111(3), pp. 588–616.

St Jacques, P.L., Conway, M.A., Lowder, M.W. and Cabeza, R. 2011. Watching my mind unfold versus yours: An fMRI study using a novel camera technology to examine neural differences in self-projection of self versus other perspectives. *Journal of Cognitive Neuroscience* 23(6), pp. 1275–1284.

Wing, E.A., Marsh, E.J. and Cabeza, R. 2013. Neural correlates of retrieval-based memory enhancement: An fMRI study of the testing effect. *Neuropsychologia* 51(12), pp. 2360–2370.
